# Does Cannabis Smoke Cause Interstitial Lung Disease?

**DOI:** 10.3390/jcm14145054

**Published:** 2025-07-16

**Authors:** Mario Bisconti, Paola Martucci, Adele Minutillo, Alessandra Palma Modoni, Raffaella Giacobbe, Maria Concetta Rotolo, Francesco Sollitto, Domenico Loizzi, Nicoletta Pia Ardò, Senia Trabucco, Salvatore Zaccaria, Paolo Fellini, Salvatore Talamo, Giuseppe Marulli, Angela De Palma

**Affiliations:** 1 Unit of Pneumology, “Vito Fazzi” Hospital, 73100 Lecce, Italy; drbiscontimario@gmail.com (M.B.); alepalmamodoni@libero.it (A.P.M.); stalamo@alice.it (S.T.); 2 Unit of Bronchial Endoscopy, “Antonio Cardarelli” Hospital, 80131 Naples, Italy; paola.martucci@aocardarelli.it (P.M.); raffaella.giacobbe@aocardarelli.it (R.G.); 3 National Centre on Addiction and Doping, Istituto Superiore di Sanità, 00161 Rome, Italy; adele.minutillo@iss.it (A.M.); mariaconcetta.rotolo@iss.it (M.C.R.); 4 Unit of Thoracic Surgery, Department of Surgery, University of Foggia, 71122 Foggia, Italy; francesco.sollitto@unifg.it (F.S.); nicoletta.ardo@gmail.com (N.P.A.); 5 Unit of Pathology, Department of Precision and Regenerative Medicine and Ionian Area, University of Bari “Aldo Moro”, 70124 Bari, Italy; xeniatrabucco@hotmail.it; 6 Unit of Cardiac Surgery, “Vito Fazzi” Hospital, 73100 Lecce, Italy; cardiochirurgia.polecce@asl.lecce.it (S.Z.); paolo.fellini@asl.lecce.it (P.F.); 7 Department of Biomedical Sciences, Humanitas University, 20072 Pieve Emanuele, Milan, Italy; giuseppe.marulli@hunimed.eu; 8 Thoracic Surgery, IRCCS Humanitas Research Hospital, 20089 Rozzano, Milan, Italy; 9 Unit of Thoracic Surgery, Department of Precision and Regenerative Medicine and Ionian Area, University of Bari “Aldo Moro”, Piazza Giulio Cesare 11, 70124 Bari, Italy

**Keywords:** interstitial lung disease, drugs, cannabis smoke, cannabinoids, broncho-alveolar lavage, bronchoscopy, lung tissue, pneumothorax, biomarker

## Abstract

**Background/Objectives**: The correlation between drugs and interstitial lung disease (ILD) is reported, but the presence of the substances of abuse in the lung as a cause of disease has never been proved. In this observational study, our aim was to evaluate a possible correlation between ILD radiological findings and cannabinoids presence in broncho-alveolar lavage (BAL) or in resected lung tissue in patients with a history of cannabis smoke. **Methods**: Data of patients with ILD chest CT scan findings and history of drug use, submitted to BAL (Group 1), or to lung apex removal for pneumothorax (Group 2), were retrospectively collected. In both groups, drug presence was investigated. A subgroup of Group 1 was checked for the concomitant presence in blood. Fisher’s test was used to study the association between the detection of the drug and ILD. **Results**: In Group 1, cannabinoids were present in 12/26 (46.2%) BAL samples. ILD emerged on chest CT in 75% of the cannabinoid-positive and in 20% of the cannabinoid-negative BAL samples (*p* = 0.0299). In the subgroup, the patients who tested positive for cannabinoids/cocaine on BAL were 55.6%; 0% were positive only on blood (*p* = 0.0294). In Group 2, cannabinoids were present in 10/15 (66.7%) specimens. ILD was evident, respectively, in 40% and in 0% of the patients with cannabinoid-positive and cannabinoid-negative surgical specimens (*p* = 0.2308). **Conclusions**: The prevalence of ILD in patients with cannabinoid-positive BAL and in those with cannabinoid-positive surgical specimens suggests that ILD could be caused by cannabis smoke. The non-concomitant presence of substances in BAL and in blood advocates the diagnostic usefulness of searching for the drug in the target organ.

## 1. Introduction

Respiratory diseases caused by illicit drugs (Re.Di.D.) have remote origins, but most reports regarding the respiratory system began in the second half of the twentieth century: lung emphysema, pulmonary arterial hypertension, interstitial lung disease (ILD), and others [1]. However, to date, it appears difficult to define the epidemiological data of the various drug-related diseases, because not all subjects who take illicit drugs report using them, which is essential for formulating an appropriate diagnosis.

Illicit drug use may cause damage to the lung occurring from either systemic or inhalational consumption: drugs administered by intravenous route may result in foreign body granulomas with vasculitis and fibrotic reaction, while inhaled drugs may promote airway injury and the stimulation of nonspecific inflammation processes, potentially leading to develop chronic ILD; in fact, chest computed tomography (CT) findings of cocaine-induced granulomatosis and pulmonary fibrosis have been reported [1]. Likewise, in 22 cocaine users, chest CT evidence of ILD was observed in a subgroup of 8 (36.4%) [2]. Furthermore, interstitial pneumonia, interstitial fibrosis, and pulmonary arterial hypertension due to substances of abuse (cocaine and cannabis) have been described [3,4,5,6]. On the other hand, one review and two population and cohort studies have failed to provide consistent evidence regarding the negative impact of cannabis smoke on the respiratory system, as symptoms were not greater than those observed in tobacco smokers and they were not additive [7,8,9].

Illicit drugs are searched for in urine, blood, and hair. In clinical practice, time correlation between taking the substance and the onset of a respiratory disease can suggest the substance’s responsibility when it is detected in blood or urine, although the diagnosis is only presumptive if it is not found in the lung tissue where the disease occurs.

Thus, in order to define a more suitable path to reach an appropriate clinical-radiological diagnosis, in two of our previous studies, we developed a method to identify cannabinoids in broncho-alveolar lavage (BAL) and we searched and found cannabinoids in the lung tissues of cannabis smokers operated for pneumothorax [10,11]. Moreover, in another of our studies, we demonstrated the presence of bullous emphysema in lung tissues positive for cannabinoids, underlining the role of cannabis in the pathogenesis of pneumothorax [12].

The correlation between drugs and interstitial lung disease (ILD) is reported, but the presence of the substances of abuse in the lung as a cause of disease has never been proved. Consequently, focusing our attention on ILD, in this observational study, our aim was to evaluate a possible correlation between chest CT evidence of ILD and cannabinoids presence in BAL or in resected lung tissue in patients with a history of cannabis smoke. A secondary aim was to investigate the usefulness of searching for the drug in the target organ of the disease: in BAL and not in blood. This study may open the way to the study of drug-related pathogenic mechanisms, which are still unknown.

## 2. Materials and Methods

The study was conducted in accordance with the Declaration of Helsinki (as revised in 2013) and was approved by the Ethics Committee of the “Vito Fazzi” Hospital, Lecce, Italy (6 June 2019/n. 33).

Data of patients with ILD radiological findings at chest CT scan and history of drug use, submitted to BAL through bronchoscopy (Group 1), or lung apex removal (apicoectomy) for pneumothorax (Group 2), were retrospectively collected from 2016 to 2019. We recruited subjects who reported illicit drugs use in their clinical history; however, in cases where the intake was not spontaneously declared and a possible etiological factor responsible for respiratory symptoms did not emerge, after the exclusion of common etiological factors, we suspected the use of illicit drugs and we conducted the so-called “voluptuary anamesis”, which paved the way, allowing us to identify other users. The collection of the voluptuary anamesis implies the following: to establish a confidential relationship with the subject; to collect the anamnesis in a private, undisturbed environment; to conduct a “humanized” and not “technicized”anamnesis; to remember the doctors’ obligation of professional secrecy; to explain that to cure his/her illness it is necessary to discover the casual factor; and to ask the subject if he or she has ever taken any drugs of abuse.

Inclusion criteria were age ≥ 18 years; smokers or former smokers of cannabis; patients with diseases requiring chest CT and BAL or lung tissue resection for pneumothorax; chest CT evidence of ILD; and the signing of written informed consent for the toxicological examination of BAL, lung tissue, and blood.

Elements suggesting to collect the voluptuary anamnesis to identify the drug user were exclusion of etiological factors commonly causing the observed disease, some typical characteristics of drug users, and report of accidents with unclear dynamics responsible for traumatic injuries; in these cases, we explained the study and invited the patient to sign the informed consent to participate in the study.

Patient recruitment took place as they were admitted to the hospital based on their acceptance to participate in the study by signing the written informed consent. Patients were asked questions about the intake of cocaine, heroin, the number of tobacco and cannabis cigarettes smoked per day, the last intake before BAL or pneumothorax, and symptoms.

In Group 1, we included patients with diseases requiring chest CT and BAL, and in Group 2, those who needed chest CT and surgery for pneumothorax. In both groups, cannabinoids presence in BAL or in lung tissue, respectively, was investigated. A subgroup of Group 1 was checked for the concomitant presence in blood. In this subgroup, patients who gave their consent allowing us to take and examine even a blood sample were recruited.

### 2.1. Group 1

In Group 1, three samples were taken from each patient undergoing BAL: one was sent for microbiological examination, one to the Unit of Pathology for standard cytological examination, and the other one was frozen (−18 °C) and sent to the National Centre on Addiction and Doping for the toxicological examination and identification of cannabinoids and other drugs in BAL [10].

#### Subgroup

A subgroup of patients of Group 1 underwent blood sampling, too, for toxicological examination, to be compared with that of BAL.

### 2.2. Group 2

Two specimens were taken from each patient submitted to lung apicoectomy (Group 2): one was sent to the Unit of Pathology for standard pathological examination, and the second was frozen (−18 °C) and sent to the National Centre on Addiction and Doping for the toxicological examination and identification of cannabinoids in lung tissue [11].

### 2.3. Statistical Analysis

Quantitative variables were summarized as mean and range.

Nominal variables were reported as counts and percentages.

Fisher’s exact test was used to study the association between the detection of drugs in BAL or in lung tissue and chest CT evidence of ILD (Groups 1 and 2) and between the presence of drugs in BAL and in blood (subgroup of Group 1); *p*-values < 0.05 were considered significant.

Statistical analyses were performed using RStudio (R v3.6.2, Dark and Stormy Night).

## 3. Results

### 3.1. Group 1

Group 1 consisted of 26 patients, 24 males and 2 females, mean age 38.42 years (range 18–56 years), who underwent BAL and chest CT (Table 1). The average duration of exposure to cannabis smoke was 15 years. The males were all smokers of tobacco and cannabis, and 10 were cocaine and 6 heroin users, too. One of the two female patients was a passive cannabis smoker, and the other one was a smoker of tobacco and cannabis and a cocaine user.

Table 1 reports the characteristics of the patients submitted to BAL, the results of the toxicological examination on BAL, and the chest CT findings.

Twelve subjects (46.2%) tested positive for cannabinoids on BAL, four for cocaine; in ten, a negative toxicological result was found.

At chest CT, ILD was detected in 9/12 (75%) cases with cannabinoid-positive BAL and in 2/10 (20%) with cannabinoid-negative BAL. The association between cannabinoids in BAL and ILD detected at chest CT was statistically significant (*p* = 0.0299; 95% C.I. [1.189–157.526]).

**Figure 1:**
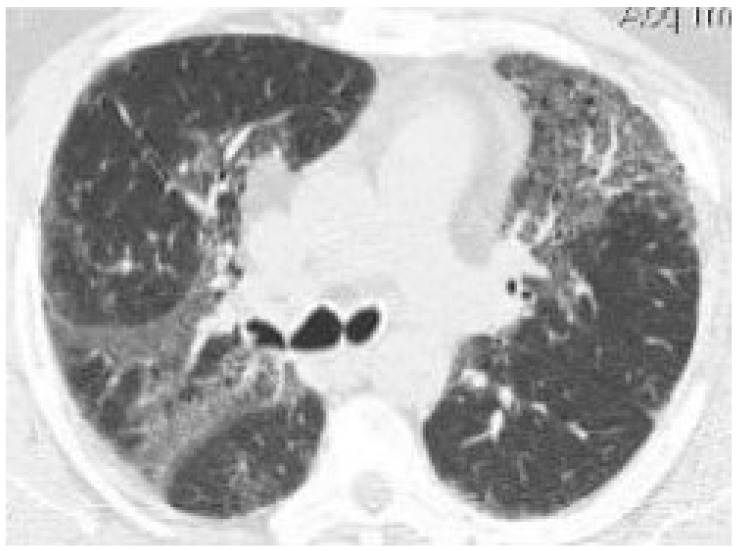
Chest CT of one of the patients with cannabinoid-positive BAL showing bilateral ground-glass opacities (GGO), demarcated by areas of normal lung parenchyma, creating a “mosaic” lung pattern; in the context of the GGO, thickening of the lung interstitium with formation of microcysts can be seen.

In the nine patients with cannabinoid-positive BAL, we observed ground-glass opacities in four (44.4%), tree-in-bud sign in two (22.2%), bilateral micronodules in one (11.1%), and consolidations in one (11.1%) (Figure 1).

In the two patients with cannabinoid-negative BAL, we observed ground-glass opacities in both (100%), tree-in-bud sign in none (0%), bilateral micronodules in none (0%), and consolidations in none (0%).

We found ILD at chest CT in 3/4 (75%) patients positive for cocaine on BAL. In particular, we found consolidations in two (66.6%), ground-glass opacities in one (33.3%), tree-in-bud sign in one (33.3%).

The female patient, a passive smoker of 2–3 cannabis cigarettes per day, smoked by her partner for 7 years, had hemoptoe, hematuria, proteinuria, and cANCA positivity on serum. Chest CT showed ground-glass opacity, and BAL was positive for cannabinoids at toxicological examination. Alveolar cytogram showed a carpet of macrophages with coarse deposits of hemosiderin, positive Perls histochemical staining, and neutrophil granulocytes. The histological examination of two renal biopsy samples highlighted focal segmental glomerulonephrosis, a probable evolutionary outcome of a previous ANCA-associated pauci-immune vasculitic form, in remission.

#### Subgroup

A subgroup of Group 1 was checked for the concomitant presence of cannabinoids in blood. The subgroup consisted of thirteen patients (not included in Table 1), all males (Table 2), mean age 34.38 years (range 22–42 years), smokers of tobacco and cannabis; two were cocaine users, too. They underwent toxicological examination, each at the same time, on BAL and on blood. It emerged that 9/13 (69.2%) were positive for cannabinoids/cocaine on BAL; of these, 4/13 (30.8%) were positive for cannabinoids on BAL and blood simultaneously, suggesting a transfer of the substance from the lung to the blood.

Furthermore, excluding the 4 patients in whom the drug was present simultaneously in BAL and in blood, 5/9 (55.6%) of the remaining ones showed the substances in BAL and 0/9 (0%) in blood and the difference was statistically significant (*p* = 0.0294; 95% C.I. [1.248–∞]).

### 3.2. Group 2

Group 2, including the patients who underwent lung apicoectomy and chest CT, was composed of 15 males, tobacco and cannabis smokers, and 1 multi-drug user (cannabis, cocaine, and heroin), mean age of 26.86 years (range: 18–51 years). The average duration of exposure to cannabis smoke was 9.5 years.

Table 3 reports the characteristics of the patients submitted to lung apicoectomy for pneumothorax, the results of the toxicological and histological examination on the surgical specimen, and the chest CT findings.

Cannabinoids were present in 10/15 (66.7%) specimens.

On chest CT, ILD emerged in 4/10 (40%) cases with cannabinoid-positive surgical specimens, and in 0/5 (0%) with cannabinoid-negative surgical specimens (*p* = 0.2308; 95% C.I. [0.347–∞]). Among the four cannabinoid-positive patients with ILD, we observed ground-glass opacities in one (25%), ground-glass opacities and consolidations together in two (50%), and micronodules in one (25%). At the histological examination, the lung tissue of these four cannabinoid-positive patients with ILD on chest CT scan was characterized by interstitial fibrosis and DIP-like reaction, more specifically, the patient with ground-glass opacities had interstitial fibrosis; the two patients with ground-glass opacities and consolidations and the patient with micronodules all three had DIP-like reaction and two interstitial fibrosis.

**Figure 2:**
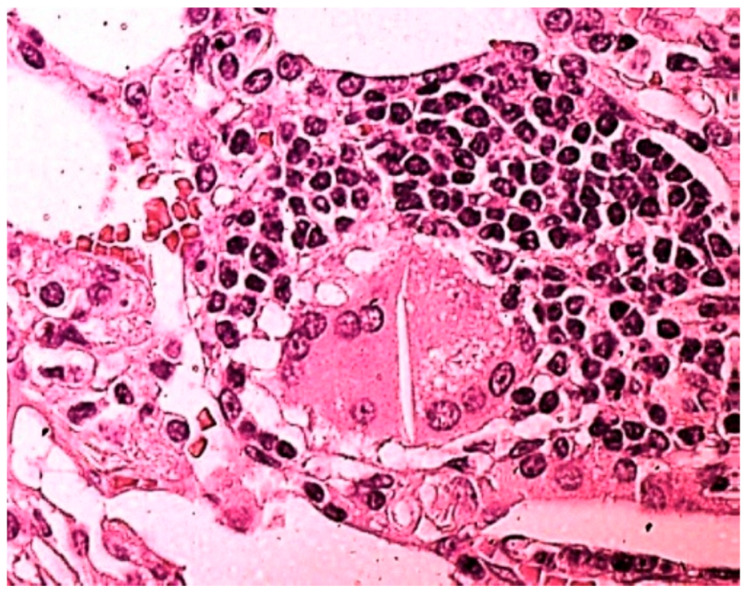
Magnification of 250×, hematoxilin–eosin staining (HE): elongated needle-like inclusions crossing almost the entire body of a multinucleated cell. The evaluation under a polarized light microscope highlighted the birefringence of the needle-like inclusions and crystals with a morphology of organic fibers, such as cellulose, thus suggesting the diagnosis of pulmonary fibrosis and granulomatosis from cellulose-cut cocaine.

The patient in Figure 2, not included in Table 3 because at that time we had not developed yet the technique of searching for drugs in BAL [10] and lung tissue [11], smoked 20 tobacco cigarettes a day for 20 years; he had been snorting cocaine once a day for 10 years until the day before surgery; urine toxicological examination resulted positive for cocaine. Chest CT showed thickening of the peribronchovascular and septal interstitium, right apical thickening, diffuse micronodulia, and mediastinal lymphadenopathy. In BAL, there were CD4 lymphocytic alveolitis, increased neutrophil and eosinophil counts, numerous giant cells, mast cells, and birefringent bodies. The histological examination of the lung tissue showed interstitial fibrosis, granulomatous giant cell foci characterized by epithelioid cells, Langhans-type giant cells with cytoplasmic inclusions, attributable to asteroid bodies, and absence of caseous necrosis. The evaluation of the slide under a polarized light microscope highlighted the birefringence of needle-like inclusions (Figure 2), thin and elongated crystals between 10 and 40 microns in size, and with a morphology of organic fibers, such as cellulose, thus suggesting the diagnosis of pulmonary fibrosis and granulomatosis from cellulose-cut cocaine.

## 4. Discussion

ILDs are a heterogeneous group of disorders characterized by the development of lung fibrosis. The pathogenesis implicates a series of inflammation and fibrosis disrupting the interstitial bed and changing the lung parenchyma. Causing factors can be exogenous or endogenous. ILDs without identifiable causes are considered idiopathic.

ILD can be classified on the basis of histological and radiological parameters.

The main alterations concerning the interstitium and air space of the lung are ground-glass opacities, reticular markings, thickened airways, diffuse centrilobular nodules, pulmonary fibrosis, desquamative interstitial pneumonia (DIP), and others [13].

Among the exogenous causes of ILD, illicit drugs have been reported [1,2,3,13,14,15,16,17,18].

The substance of abuse most used by our patients was cannabis.

In Group 1, ILD emerged at chest CT in 75% of the subjects with cannabinoid-positive BAL and in 20% of the subjects with cannabinoid-negative BAL. This statistically significant difference (*p* = 0.0299) suggests the possible role of cannabinoids in causing ILD. Furthermore, the respiratory damage of second-hand tobacco smoke is known, but not that of cannabis smoke. Since cannabis is the most widespread psychotropic substance in the world, the observation of a patient passive smoker, who had a cannabinoid-positive BAL from second-hand smoke, suggests further studies, even in subjects who are not active users.

Moreover, in Group 1, among the nine patients with chest CT signs of ILD and cannabinoid-positive BAL, the main cannabis-induced organ lesions were ground-glass opacities in 44.4%, tree-in-bud sign in 22.2%, bilateral micronodules in 11.1%, and consolidations in 11.1%. Thus, this evidence would suggest, in cases where these radiological patterns are found, to better investigate the possible use of illicit drugs.

In Group 2, ILD was evident at chest CT in 4/10 (40%) patients with cannabinoid-positive surgical specimens, and in 0/5 (0%) with cannabinoid-negative surgical specimens. In this case, the association between cannabinoids in lung tissue and ILD that emerged from chest CT was not statistically significant (*p* = 0.2308); however, the complete absence of ILD radiological signs in the patients with cannabinoid-negative surgical specimens further suggests a role of cannabinoids in favoring ILD. Nevertheless, at histological examination, the lung tissue of the four cannabinoid-positive patients with ILD on chest CT scan was characterized by interstitial fibrosis and DIP-like reaction, thus suggesting a possible correlation among cannabinoid-positive lung tissue, pathological findings of ILD on the same tissue, and chest CT evidence of ILD.

In addition, in Group 2, among the four cannabinoid-positive patients with chest CT signs of ILD, we observed ground-glass opacities, consolidations, and micronodules, again suggesting, in case of these radiological findings, to consider a possible history of illicit drug use.

Moreover, the difference in chest CT findings of ILD, in 75% of the patients of Group 1 subjected to BAL, compared to 40% of those of Group 2 operated for pneumothorax, could be explained by the longer time of exposure to cannabis smoke. In fact, in the patients of Group 1, who were older, with a mean age of 38.42 years, the average duration of exposure to cannabis smoke was longer, 15 years, thus justifying the greater number of subjects with radiological evidence of ILD. In Group 2, composed of younger patients, mean age 26.86 years, the shorter average time of exposure to cannabis smoke, 9.5 years, may explain the smaller number of subjects with chest CT detection of ILD. This suggests the harmful action of cannabis in relation to the time of exposure.

Even the cellularity that emerged in the histology of surgical specimens in patients of Group 2 supports the hypothesis of the damaging action of cannabis in causing ILD. In fact, the respiratory system is subjected to the chronic irritative insult of tobacco and cannabis smoke, associated with the presence of pollutants responsible for the cellularity present in histological samplings. In the damaged tissue, neutrophils and monocytes are activated, which release toxic factors: proteases, elastase, and collagenase. The chronic irritative stimulus would favor the formation of emphysematous bullae earlier in cannabinoid-positive lung tissue [11]. The presence of macrophages or lymphoplasma cells or foreign body giant cells (Table 3) supports the action of cannabinoids in promoting the nonspecific chronic inflammatory process, with secondary lysis of the interalveolar septa, site of the chronic flogistic process, with interstitial fibrosis/DIP.

In our patients, cellularity involved in a nonspecific chronic inflammatory process was present in 9/10 (90%) patients with cannabinoid-positive lung tissue and in 0/5 (0%) patients with cannabinoid-negative lung tissue. In cannabinoid-positive patients and in cannabinoid-negative ones, respectively, we observed macrophages in 6/10 (60%) and in 0/5 (0%), lymphoplasma cells in 2/10 (20%) and in 0/5 (0%), and foreign body giant cells in 3/10 (30%) and in 0/5 (0%), thus defining, as far as we know, for the first time in the literature, this phenotype associated with cannabinoids in surgical specimen.

Other studies suggest that marijuana causes pulmonary fibrosis [19,20]. In an autopsy study, the intensity of macrophage infiltration appears to be related to the dose of marijuana taken [19].

Desquamative interstitial pneumonia (DIP) is reported in users of large quantities of marijuana [20,21]. In our study, DIP-like reaction (Table 3) was found in 5/10 (50%) patients with cannabinoid-positive lung tissue and in 1/5 (20%) with cannabinoid-negative lung tissue. We observed the accumulation of macrophages in the five cannabinoid-positive patients with DIP-like reaction; absence in the only cannabinoid-negative patient with DIP-like reaction (patient n. 14, Table 3). Our finding suggests the responsibility of cannabis in causing DIP, especially taking into consideration the young age of the patients (24–27 years) (Table 3). Indeed, in DIP from other causes, the average age of onset of symptoms is between 40 and 60 years [22,23]. Not only can drugs be responsible, but also additives, adulterants, and cutting substances, such as cellulose, talc, and starch [2,6,24], as in the patient in Figure 2: the research in the granuloma was crucial for a correct etiological diagnosis.

Among the manifestations caused by substances of abuse, ground-glass opacities have been described. In a study of cocaine users, in a subset of patients with chest CT evidence of ILD, ground-glass opacities were present in 100% of the cases, consolidations in 50%, and nodular formations and tree-in-bud pattern in 12.5% [2]. This study, based on statistical–radiological data, suggests the responsibility of cocaine, and ours, based on statistical–radiological–toxicological data, further supports it. Indeed, in the four patients of Group 1 with cocaine-positive BAL (Table 1), chest CT showed ground-glass opacities in 25%, consolidations in 50%, and tree-in-bud pattern in 25% of cases.

To formulate an etiological diagnosis, the substance of abuse is usually searched for in the blood, but its diagnostic reliability has not yet been proven. Thus, to verify the usefulness of searching for the substance of abuse in the blood or in the organ where the disease is located, we studied a subgroup of patients of Group 1. In the subgroup, eliminating the confounding elements, represented by the four patients who simultaneously presented the substance in BAL and in blood (thus suggesting a transfer of the substance from the respiratory tract to the blood), 55.6% of the remaining patients resulted positive in BAL and none of them in blood, with a statistically significant difference (*p* = 0.0294). Although it was expected to find the substance in the blood of all the patients, this was not the case, underlining the usefulness of searching for the drug in the organ where the disease is located, thus in BAL and not in the blood, to formulate a reliable etiological diagnosis.

Lastly, our enrolled patients did not have any chronic diseases, infectious factors, nor were they users of other medications such as fibrotizing drugs that might have been responsible for the documented ILD.

This study has some limitations: the relatively limited number of patients and consequent power limitation due to the small sample size, especially the cannabinoid-negative cases, which could have been a confounding factor in the comparative analysis between positive and negative cases, causing potential biases; the lack of a true control group; the retrospective design of the study; and difficulties in recruiting patients who were reluctant to provide informed consent for the toxicological examination of their BAL or lung tissue or blood. Most of our patients were active tobacco smokers, which could have been a confounding or contributing factor in the pathologic findings. However, to the best of our knowledge, there are no studies demonstrating that tobacco smoking is associated with the direct development of ILD. Furthermore, co-exposure to other drugs might have some importance, but the major role should be attributed to the drug identified in the examined biological samples. The simple anamnestic data of substance abuse is relative. To ascribe a major role to the drugs used and not identified in biological samples is only a hypothesis of responsibility, as reported in other previous studies where only the anamnesis acted as an etiological diagnostic guide. Thus, not having found other responsible etiological factors, we should ascribe responsibility to cannabis in the cases in which it has been found in biological samples.

## 5. Conclusions

In conclusion, in our study, the prevalence of ILD in patients with cannabinoid-positive BAL and in those with cannabinoid-positive lung surgical specimens suggests a possible causative role of cannabis smoke in ILD and potentially defines a cannabis-related ILD.

Moreover, the non-concomitant presence of substances of abuse in BAL and in blood advocates the diagnostic usefulness of searching for the drug in the target organ.

Since now, the diagnosis was based only on the anamnestic data of substance abuse, while our study supports, in clinical practice, the indication for toxicological examination of the lung.

In addition, our study suggests looking for substances of abuse in other organs as well. Finally, further studies on BAL and lung tissues to identify biomarkers, drug activities, or drug-induced toxicity and metabolism are recommended. In fact, our study is not a point of arrival, but a starting point, as it opens the way to further studies, such as metabolomics research, to analyze the action of cellular damage and the time of permanence of these illicit substances in the bronchi and lung tissues.

## Figures and Tables

**Table 1:** Characteristics of patients of Group 1, submitted to BAL; results of toxicological examination on BAL, and chest CT findings.

Patient N.	Gender	Age (Years)	Type of Smoke/ Substance of Abuse *	Smoke/Abuse Duration (Years)	Cannabis Cigarettes Smoked or Other (per Day)	Last Intake Before BAL	Toxicological Results on BAL *	Chest CT Findings *
1	M	43	T/C	20/15	20/n.d.	1 day/5 days	THC, CBN, CBD	a,b
2	F	42	C (passive smoker)	7	2–3	4 months	THC, CBN, CBD	a
3	M	56	T/C/H	46/46/46	30/3/3	5 min/4 years/ 4 years	Negative	c
4	M	44	T/C	34/n.d.	20/1	5 min/2 months	Negative	d
5	M	40	T/C	20/10	20/4 per week	5 min/2 months	Negative	e
6	M	55	T/C/H (i.v.)	41/40/17	20/1/n.d.	5 min/3 days/ 20 years	THC, CBN, CBD	c
7	M	44	T/C	26/n.d.	20/occasional	5 min/6 months	Negative	a
8	M	45	T/C/Co	31/25/n.d.	20/1/n.d.	5 min/5 days/n.d.	THC,CBN,CBD	f
9	M	44	T/C	19/n.d.	20/occasional	5 min/3 months	Negative	c
10	M	18	T/C	4/1	n.d./n.d.	5 min/20 days	Cocaine, Benzoylecgoine	g
11	M	46	T/C/Co/H	32/30/n.d./n.d.	30/3–4/n.d./n.d.	5 min/n.d./ 8 months/8 months	Negative	c
12	M	43	T/C/Co	20/15/20	20/n.d./n.d.	1 day/5 days/5 days	THC, CBN, CBD	b
13	M	47	T/C/Co	20/15/10	20/n.d./n.d.	1 day/20 days/1 day	Cocaine, Benzoylecgoine	a,b
14	M	22	T/C/Co	10/15/10	20/occasional/occasional	1 day/5 days/5 days	Cocaine, Benzoylecgoine	i
15	M	46	T/C/Co/H	32/16/n.d./n.d.	30/3–4/n.d./n.d.	1 day/8 months/ 8 months/8 months	Negative	c
16	M	46	T/C/Co/H	32/16/n.d./n.d.	30/3–4/occasional/ occasional	1 day/8 months/ 8 months/8 months	Cocaine, Benzoylecgoine	g,h
17	M	33	T/C	14/15	-	-	THC, CBN, CBD	h
18	M	33	T/C	15/15	20	-	Negative	tracheal stenosis
19	M	39	T/C	14/19	20/5	1 month	Negative	a
20	M	18	T/C	3/2	20/2	-	THC, CBN, CBD	c
21	M	22	T/C/Co/K	7/6/6/6	20/5	3 months	Negative	bronchitis
22	F	40	T/C/Co	29/19/1	2–3 per week/2–3	20 days/2 months	THC, CBN, CBD	c
23	M	20	T/C	6/6	20/2	1 day/1 day	THC, CBN, CBD	e
24	M	49	T/C/Co/H	37/36/15/10	40/20	-	THC, CBN, CBD	pleural effusion
25	M	33	T/C	-	-	-	THC, CBN, CBD	a
26	M	31	T/C/Co	17/16/16	31/1–2	15 days	THC, CBN, CBD	a

* T = tobacco; C = cannabis; K = ketamine; Co = Cocaine; H = heroin; i.v. = intravenously; n.d. = not declared. THC = delta-9-tetrahydrocannabinol; CBN = cannabinol; CBD = cannabidiol. (a) ground-glass opacities; (b) tree-in-bud sign; (c) bilateral pulmonary opacities; (d) pulmonary emphysema; (e) pulmonary abscess; (f) pulmonary bilateral micronodules; (g) bullous emphysema; (h) consolidations; (i) no lesions.

**Table 2:** Results of the patients of the subgroup of Group 1, users of substances of abuse and subjected at the same time to sampling for toxicological examination on BAL and on blood.

Patient n.	Gender	Age (Years)	Toxicological Results on BAL *	Toxicological Results on Blood *
1	M	34	THC, CBN, CBD	Negative
2	M	30	Negative	Negative
3	M	40	THC, CBN, CBD	THC, CBN, CBD
4	M	28	Negative	Negative
5	M	38	THC, CBN, CBD	THC, CBN, CBD
6	M	39	Negative	Negative
7	M	31	Negative	Negative
8	M	37	THC, CBN, CBD	THC, CBN, CBD
9	M	36	THC, CBN, CBD	THC, CBN, CBD
10	M	40	THC, CBN, CBD	Negative
11	M	42	THC, CBN, CBD	Negative
12	M	22	Cocaine, Benzoylecgoine	Negative
13	M	30	Cocaine, Benzoylecgoine	Negative

* THC = delta-9-tetrahydrocannabinol; CBN = cannabinol; CBD = cannabidiol.

**Table 3:** Characteristics of patients of Group 2, operated for pneumothorax: toxicological and histopathological results on lung tissue and chest CT findings.

Patient N.	Gender	Age (Years)	Type of Smoke/ Substance of Abuse *	Tobacco Cigarettes Smoked (per Day)	Cannabis Cigarettes Smoked (per Day)	Cannabis Smoke/Abuse Duration (Years)	Toxicological Results on Lung Tissue *	Pathological Findings *	Cellularity *	Chest CT Finding *
1	M	24	T/C	10	1	9	THC, CBN, CBD	a	d	-
2	M	27	T/C	20	1 per month	n.d.	THC, CBN, CBD	a	c	f
3	M	27	T/C	20	1	11	THC, CBN, CBD	b	c	f,h
4	M	24	T/C	15	1	9	THC, CBN, CBD	a,b	c,d	-
5	M	39	T/C	10	1 per month	15	Negative	no fibrosis	-	-
6	M	27	T/C	20	1	13	THC, CBN, CBD	a	e	-
7	M	27	T/C	20	1	11	THC, CBN, CBD	a,b	c	f,h
8	M	18	T/C	5	1 per month	3	Negative	a	none	-
9	M	24	T/C	15	1	9	THC, CBN, CBD	a,b	c,d	-
10	M	27	T/C	3–5	1	10	THC, CBN, CBD	a,b	c	g
11	M	19	T/C	7–9	2	4	THC, CBN, CBD	a	e	-
12	M	25	T/C	15	1 per month	7	Negative	-	none	-
13	M	25	T/C	10	1	9	Negative	a	-	-
14	M	51	T/C/Co/H	50	1	20	Negative	b	-	-
15	M	19	T/C	5	1 per month	3	THC, CBN, CBD	a	none	-

* T = tobacco; C = cannabis; Co = cocaine; H = heroin; n.d. = not declared. THC = delta-9-tetrahydrocannabinol; CBN = cannabinol; CBD = cannabidiol. (a) interstitial fibrosis; (b) DIP-like reaction; (c) macrophages; (d) giant cells; (e) plasma cells; (f) ground-glass opacities; (g) nodules; (h) consolidation.

## Data Availability

The datasets used and/or analyzed during the current study are available from the corresponding author upon reasonable request.

## Abbreviations

ReDiD	respiratory diseases caused by illicit drugs
ILD	interstitial lung disease
CT	computed tomography
BAL	broncho-alveolar lavage
THC	delta-9-tetrahydrocannabinol
CBN	cannabinol
CBD	cannabidiol
DIP	Desquamative interstitial pneumonia
